# Computer-aided design of metal chalcohalide semiconductors: from chemical composition to crystal structure†
†Electronic supplementary information (ESI) available. See DOI: 10.1039/c7sc03961a


**DOI:** 10.1039/c7sc03961a

**Published:** 2017-12-04

**Authors:** Daniel W. Davies, Keith T. Butler, Jonathan M. Skelton, Congwei Xie, Artem R. Oganov, Aron Walsh

**Affiliations:** a Centre for Sustainable Chemical Technologies , Department of Chemistry , University of Bath , Claverton Down , Bath BA2 7AY , UK . Email: k.t.butler@bath.ac.uk; b Science and Technology on Thermostructural Composite Materials Laboratory , International Center for Materials Discovery , School of Materials Science and Engineering , Northwestern Polytechnical University , Xian , Shaanxi 710072 , Peoples Republic of China; c International Center for Materials Discovery , School of Materials Science and Engineering , Northwestern Polytechnical University , Xian , Shaanxi 710072 , Peoples Republic of China; d Skolkovo Institute of Science and Technology , 3 Nobel Street , Moscow Region 143026 , Russia; e Moscow Institute of Physics and Technology , Dolgoprudny , Moscow Region 141700 , Russia; f Department of Materials Science and Engineering , Yonsei University , Seoul 03722 , Korea . Email: a.walsh@imperial.ac.uk; g Department of Materials , Imperial College London , Exhibition Road , London SW7 2AZ , UK

## Abstract

The standard paradigm in computational materials science is INPUT: Structure; OUTPUT: Properties, which has yielded many successes but is ill-suited for exploring large areas of chemical and configurational hyperspace. We report a high-throughput screening procedure that uses compositional descriptors to search for new photoactive semiconducting compounds. We show how feeding high-ranking element combinations to structure prediction algorithms can constitute a pragmatic computer-aided materials design approach. Techniques based on structural analogy (data mining of known lattice types) and global searches (direct optimisation using evolutionary algorithms) are combined for translating between chemical composition and crystal structure. The properties of four novel chalcohalides (Sn_5_S_4_Cl_2_, Sn_4_SF_6_, Cd_5_S_4_Cl_2_ and Cd_4_SF_6_) are predicted, of which two are calculated to have bandgaps in the visible range of the electromagnetic spectrum.

## Abstract 2

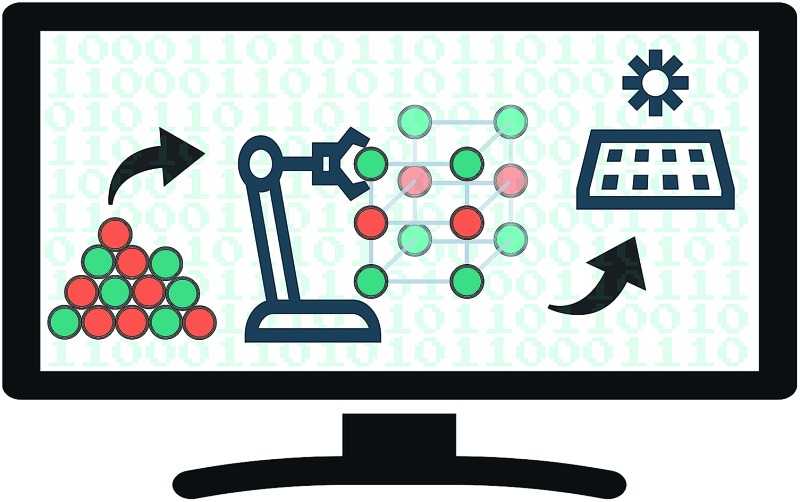
The standard paradigm in computational materials science is INPUT: Structure; OUTPUT: Properties, which has yielded many successes but is ill-suited for exploring large areas of chemical and configurational hyperspace.

## I. Introduction

The past decade has seen the emergence of many databases for computed materials properties from quantum mechanical calculations.^1^–^7^ This has made it possible to virtually screen through enormous amounts of data in the search for promising materials for energy applications such as photovoltaics,^8^–^10^ solar fuels,^11^–^15^ and thermoelectrics.^16^–^18^ Furthermore, these databases are facilitating the move towards more predictive materials design using data-mining, machine learning, and other statistical techniques to reveal hitherto undiscovered trends and rules.^19^–^29^ In order to search for Earth-abundant materials for energy applications, it is important to move beyond known materials and extend screening criteria to new compositions and structures.

There are vast areas of unexplored chemical space for inorganic compounds.^30^ Such a space is intractable to high-throughput first-principles computation, even with tremendous advances in computing power and algorithms. As such, a different approach is required to efficiently explore the search space – one that is less computationally demanding overall, but sufficiently accurate.

One modern tool that is providing impressive leaps forward in this area is machine learning (ML), a subfield of artificial intelligence that involves statistical algorithms whose performance improves with experience. A growing infrastructure of ML tools has enabled its application to complex problems in many areas of chemistry and materials science.^6^,^20^,^21^ This includes the development of models that relate system descriptors to desirable properties in order to reveal structure–property relationships,^31^ the prediction of the likelihood of a composition to adopt a given crystal structure,^32^ and the use of quantum-mechanics results as training data to extrapolate and discover new materials at a fraction of the computational cost.^29^,^33^

**Fig. 1:**
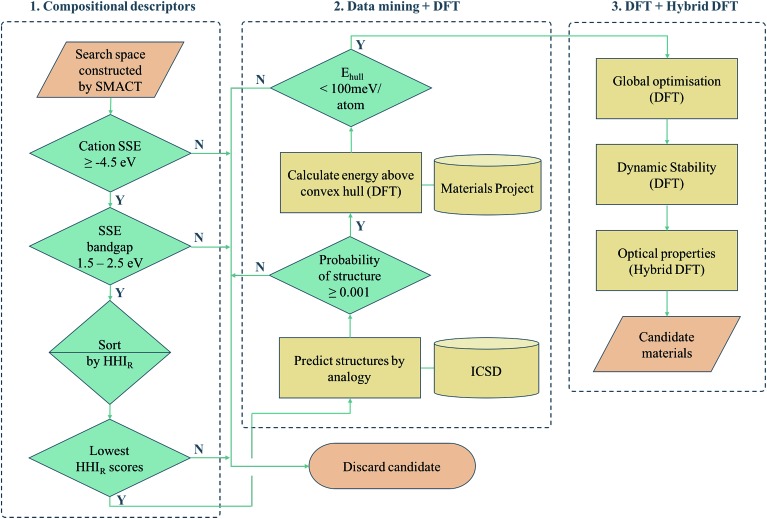
Computer-aided-design workflow used for exploring novel photoactive semiconductors. smact refers to our screening package, SSE refers to the solid-state energy scale, HHI_R_ refers to the Herfindahl–Hirschman Index for sustainability, while DFT refers to density functional theory.

Another approach is to apply a hierarchy of screening steps, based on pre-existing methods, whereby the fact that accuracy is low in initial steps is counteracted by the idea that as the size of the search space that can be screened is so large, the chance of finding a promising material at the end of the process remains high. Here we present one such workflow incorporating simple chemical descriptors, data mining from public databases, density functional theory (DFT) calculations and global structure searching algorithms (Fig. 1) to translate from a compositional search space to compounds predicted to have target properties by quantum-mechanical calculations.

We employ a multi-stage screening approach in a search for new photoactive semiconductors. While metal oxides combine many attractive properties for energy materials (*e.g.* chemical stability and low cost), they usually have bandgaps too large to absorb a significant fraction of sunlight. The formation of multi-anion compounds offers a route to modifying the electronic structure, so we consider all ternary metal chalcohalides, (*i.e.*, A_*x*_B_*y*_C_*z*_ with B = [O, S, Se, Te] and C = [F, Cl, Br, I]). As a target application, we search for materials for solar fuel generation, specifically for photoelectrochemical water splitting, where a set of well-defined screening criteria enables us to quickly narrow down the search space. Our searching methodology is built on already established and freely available materials design tools (smact, Pymatgen and uspex) and can be adapted to search for different classes of materials, in a wide range of contexts of technological interest.

## II. Results

### II.I. A_*x*_B_*y*_C_*z*_ compositional screening

There exist various compositional descriptors that enable the low-cost filtering of chemical space. One such tool is the solid-state energy (SSE) scale,^34^ which can be used to estimate the positions of the valence band maxima (VBM) and conduction band minima (CBM) of a semiconductor with respect to the vacuum level using solely the identity of the constituent ions. We employ the SSE scale to carry out our compositional screening (see Computational methods section for details).

First, the smact code^30^ is used to narrow down the ternary compound search space of roughly 32 million compositions to the chalcohalide search space of 161 000 compositions. The SSE scale is then used to screen for suitable bandgaps and band-edge positions. The A cations are restricted to those with a SSE higher than the water reduction potential (approximately 4.5 V in relation to the vacuum at pH = 0) and the bandgap window was set to 1.5–2.5 eV. The latter criterion is set to a value range higher than the free energy for water dissociation (1.2 eV), in order to compensate for the combination of loss mechanisms found in practical devices that mean a bandgap as large as 2.2 eV could be required.^35^,^36^ This results in in 7676 candidate A_*x*_B_*y*_C_*z*_ compositions with unique *x*, *y*, *z* stoichiometries.

Next, we sort the candidates by the sustainability of their constituent elements based on the Herfindahl–Hirschman Index for elemental reserves (HHI_R_).^37^ The HHI_R_ includes factors such as geopolitical influence over materials supply and price, and for a given composition can be obtained as the weighted average over the constituent elements. At this stage, because stoichiometry is variable, we consider the mean value for each A_*x*_B_*y*_C_*z*_ chemical system. The six most sustainable chemical systems according to this scale are Sn_*x*_S_*y*_X_*z*_, Cd_*x*_S_*y*_X_*z*_ and Ti_*x*_S_*y*_X_*z*_, where X = [Cl, F]. Of these, the Sn- and Cd-containing compositions are selected and Ti^3+^ compounds are excluded due to the d^1^ electronic configuration being linked to fast electron–hole recombination, and, more practically, the well-known challenges for electronic-structure modelling due to the high correlation.^38^

The HHI_R_ scores of Zn_*x*_S_*y*_X_*z*_ and Cd_*x*_Se_*y*_X_*z*_ are the next lowest in the ranking, making these the next most sustainable according to this scale. This is because Zn and Se have higher HHI_R_ scores than Ti and S respectively. These systems could be of interest for future studies in the same spirit, particularly the Zn-containing compositions due to their low toxicity. This rapid screening process based on composition alone constitutes the first phase of our overall procedure (part 1 of Fig. 1).

### II.II. From chemical composition to crystal structure

Although compositional screening is a key initial step in materials exploration, the precision with which physical properties can be predicted from chemical composition alone is limited. In order to move to the next level of accuracy and make quantitative predictions, we must introduce a three-dimensional model of the arrangement of atoms in space. To our knowledge, no compounds of the compositions identified by our screening process have yet been reported, so the crystal structures must be predicted. Crystal structure prediction is a long-standing challenge in materials science,^39^ due to the large number of degrees of freedom (lattice vectors and internal coordinates) and poor scaling with increasing system complexity.

We combine two machine learning approaches for generating candidate crystal structures from chemical composition, *viz.* (1) analogy with known crystal structures reported in crystallographic databases, and (2) direct global crystal structure searching. The first approach has a much lower computational cost, exploiting data on existing compounds, and we use this step to assess the metastability of a candidate composition. Those compounds that fall within an acceptable window of metastability are then passed to the second method, which is a more rigorous search of configurational space and allows for new structure types to be adopted.

For crystal structure prediction by analogy, we adopt the structure substitution algorithm developed by Hautier *et al.*,^40^ as implemented in the Pymatgen framework.^41^ In this method, a combination of ions are substituted onto lattice sites in known structures from the Inorganic Crystal Structure Database (ICSD).^42^ Each ion substitution is associated with a certain probability, which comes from a statistical model trained on the compounds that already exist in the ICSD. If the overall probability for a given set of substitutions is above a certain threshold, it is added to a list of possible structures. This substitution process is performed on each known crystal structure in the database.

**Fig. 2:**
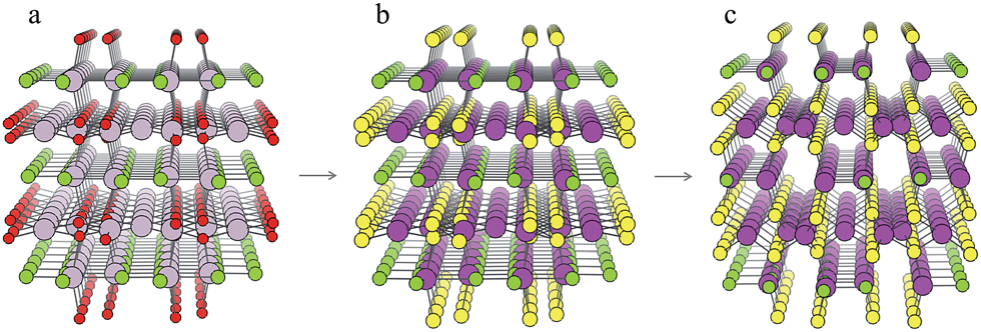
Illustration of the process of crystal structure prediction by ion substitution into existing lattice types. The Hg_5_O_4_Cl_2_ structure (a) is identified as a candidate structure for the Cd_*x*_S_*y*_Cl_*z*_ chemical system. The Hg^2+^ (grey balls) and O^2–^ ions (red balls) are replaced by Cd^2+^ (purple balls) and S^2–^ ions (yellow balls), respectively, to produce the Cd_5_S_4_Cl_2_ structure (b). Forces on the ions are then minimised using DFT with the PBEsol functional^43^ to produce the relaxed structure (c).

For each S-for-O of the four compositions, the candidate crystal structures are locally optimized using DFT calculations and the structure with the lowest energy per atom selected. Fig. 2 illustrates this process for one of the structures suggested by the algorithm for the Cd_*x*_S_*y*_Cl_*z*_ chemical system. In this case, the structure suggested is based on Hg_5_O_4_Cl_2_ due to the high probabilities associated with both Cd-for-Hg and S-for-O substitutions. Table 2 contains the chemical formulae of the four compounds deemed to be the most stable as a result of this process, along with the formulae of their parent structures in the ICSD. We next assess the thermodynamic stability of the candidate materials.

### II.III. Thermodynamic metastability

By calculating the total energies of all the competing phases of a chemical system, one can construct an energy – composition phase diagram and assess the stability of a given compound with respect to polymorphic transformations and phase separation. By creating a bounding surface between the lowest energy phases of each composition, a convex hull is constructed above which metastable compounds fall. A key value of interest for assessing the metastability of a compound is this energy above this convex hull (*E*_hull_).

Fortunately, the existence of databases of DFT total energies have all but eliminated the need for carrying out calculations for all phases of a given chemical system. Instead, one can perform calculations on new compounds using identical parameters to those used for the data in a given database, thus allowing for direct comparison of energies. Similarly, one can use the energy values in a database to construct a phase diagram and identify where on the diagram the new phase would appear. In doing so, the set of polymorphs and decomposition products that require explicit calculation can be identified. We note that it is standard to calculate such convex hulls based on internal energies, which neglect finite temperature contributions to the free energy of a compound.

**Fig. 3:**
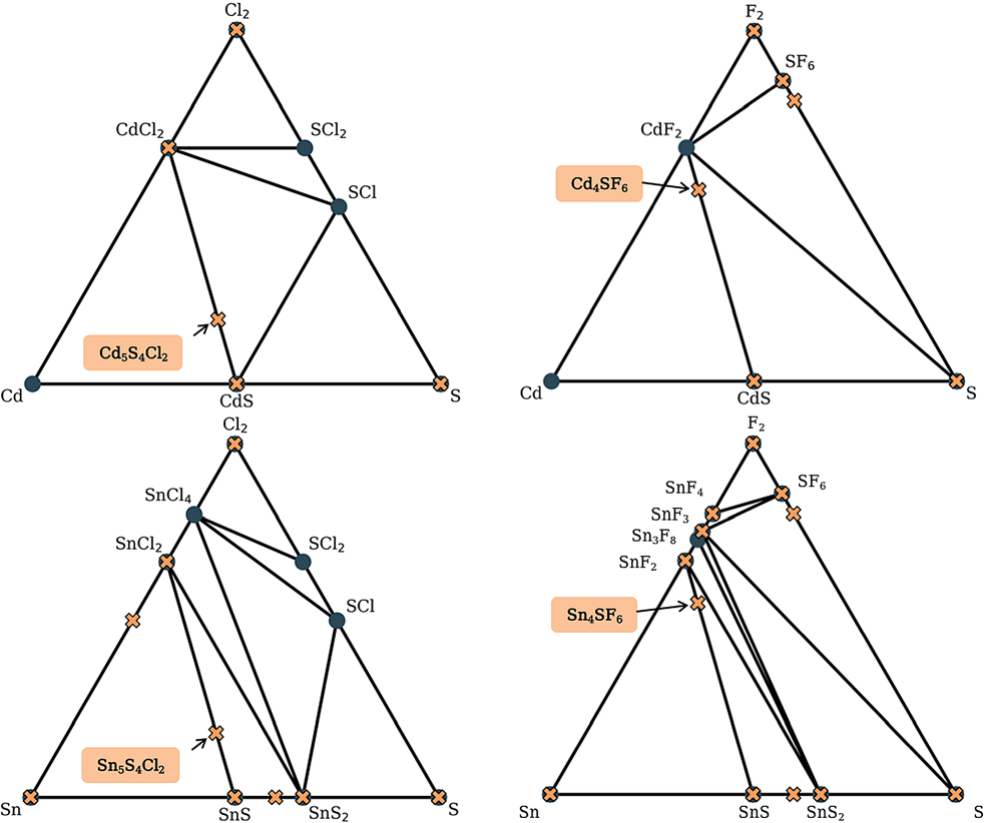
Simulated phase diagrams for the Cd–S–Cl_2_, Cd–S–F_2_, Sn–S–Cl_2_ and Sn–S–F_2_ chemical systems. Stable phases (circles) are connected by black tie-lines forming the convex hull, and unstable phases (crosses) sit above the hull. Those that are above a stable phase are unstable with respect to polymorphic changes and those above a tie-line are unstable with respect to decomposition into the stable phases at each end. The labels indicate the new phases discovered in this work.

Here, we use the Materials Project database to construct phase diagrams using the Pymatgen code,^41^ and hence identify decomposition products. As mentioned above, and as depicted in the phase diagrams in Fig. 3, it is not necessary to consider competing polymorphs as no compounds have yet been reported for these compositions. As can be seen from Table 2, all of the values of *E*_hull_ for the structures predicted by analogy lie between 18 and 97 meV per atom. Hence, all the compounds can formally be described as thermodynamically metastable at 0 K, but does this rule out their existence?

Metastable materials exist and are ubiquitous in both nature and technology. This includes obvious examples such as diamond *vs.* the lower energy allotrope of carbon, graphite, as well as classes of materials such as zeolites and metal–organic frameworks.^44^ It was recently estimated by Sun *et al.* that around half of all known inorganic materials are metastable.^25^ Whether or not the value of *E*_hull_ is enough to predict the likelihood of successful synthesis of a material is a question that has yet to be answered. In the same work by Sun *et al.*, it was shown that the likelihood of existence drops off exponentially as *E*_hull_ increases. The exact rate of the drop depends on the chemistry of the system. We use 100 meV per atom as a guiding principle for the maximum *E*_hull_, as this criteria covers approximately 90% of compounds in the Materials Project database that represent fully-characterised structures in the ICSD. The four structures found by analogy all fall within this metastability window, so they are all carried forward to the global structure searching stage.

### II.IV. Global structure search

The structure from analogy approach provides an attractive route to obtaining sensible crystal structures with reasonable energies, however it does not provide a rigorous route to obtaining the true ground state. Finding the true global ground state structure for a given chemical composition is one of the outstanding problems of theoretical chemistry. Whilst exhaustive searching of parameter space is the only way to find a guaranteed global minimum structure, this approach quickly becomes impractically expensive for even simple chemical systems. Global searching, based on evolutionary algorithms offer a solution to this problem and have had enormous success in discovering new ground state crystal structures. Here we use uspex to apply an evolutionary algorithm and perform a global structure search.

**Fig. 4:**
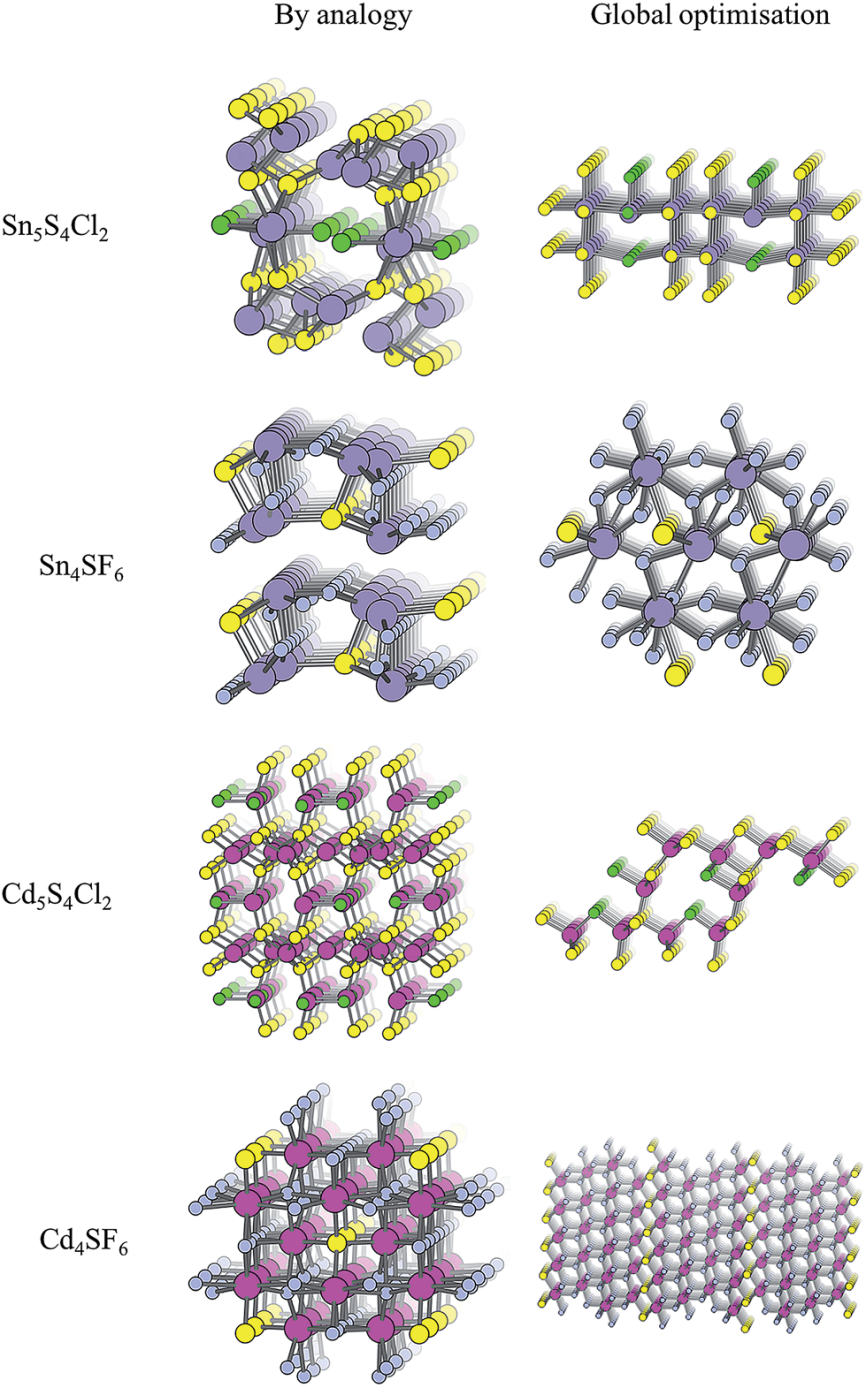
Crystal structures of the four candidate compositions as predicted by analogy through data mining of other structures and by a first-principles global structure search algorithm.

For each of the four compositions, the global structure search algorithm^45^,^46^ yields a different crystal structure to that found by analogy with known structures (Fig. 4). For each of the structures generated by the global search, there is no way in which the data-mining algorithm could have arrived at the same result. This is an intrinsic limitation of the data-mining approach, as it relies on a database of known structures and it is therefore incapable of predicting new structure types. Three of the four compounds adopt structure types that have not yet been reported, disregarding those with fractional occupancy on some lattice sites. The remaining compound, Cd_5_S_4_Cl_2_, adopts the same structure type as Li_5_BiO_5_.^47^ However, this substitution is rejected by the structure prediction algorithm on the basis that the resulting formula is not charge neutral – the structure we find is partially inverted in terms of anion/cation occupancy.

The values of *E*_hull_ for the structures predicted by global structure search are also shown in Table 2, and are universally lower than those found by analogy. While the structural analogy procedure is limited by the diversity of known structure types, the global structure search approach is restricted only by the structural complexity (number of formula units) included in the search. A holistic assessment of performance in the context of high-throughput screening must however also take into account time and resources: the data-mining algorithm takes only a few minutes to run on a desktop computer, while the global structure searching requires a supercomputing resource where around 10 000 CPU hours were needed for each material.

In addition to thermodynamic stability, another factor that cannot be ignored is dynamic stability, to ensure that the crystal structures are true local minima (and not saddle points) on the potential energy surface. Finite-displacement calculations were carried out to obtain the vibrational (phonon) frequencies of each of the compounds, and no negative-frequency (imaginary) phonon modes were found at the zone centre (*Γ* point) for any of the structures. Full details of this analysis can be found in the ESI.†

### II.V. Crystal structures and bonding environments

Table 1 contains the space groups and lattice parameters of the four minimum energy compounds identified at the end of the screening process.

**Table 1:** Structural information for the minimum energy compounds

Compound	Space group	*a* (Å)	*b* (Å)	*c* (Å)	Formula units per cell
Sn_5_S_4_Cl_2_	*Pma*2	17.529	5.771	5.817	2
Sn_4_SF_6_	*R*3	8.615	8.615	9.528	3
Cd_5_S_4_Cl_2_	*Cm*	14.507	4.212	15.631	2
Cd_4_SF_6_	*R*3*m*	3.832	3.832	37.148	3

#### Sn_5_S_4_Cl_2_

Eight Sn(ii) atoms per crystallographic unit cell adopt an octahedral environment, forming bilayers of edge-sharing SnS_5_Cl polyhedra in the *bc* plane. The polyhedra are vertex sharing at the Cl atoms, and the other two Sn atoms in the unit cell reside in the same plane as the halide ions.

#### Sn_4_SF_6_

Sn(ii) adopts both 6-and 4-coordinate environments, with space for a lone pair in each. The Sn-centred polyhedra are all vertex sharing and have either 6 F vertices (6-coordinate Sn) or 3 F vertices and 1 S vertex (4-coordinate Sn).

#### Cd_5_S_4_Cl_2_

Two Cd(ii) atoms per unit cell locate at the centre of CdS_4_ tetrahedra, and seven Cd atoms form the centre of CdS_3_Cl tetrahedra. The other two Cd atoms form trigonal bipyramids with 3 S and 2 Cl vertices. All of the polyhedra are vertex sharing bar one of the trigonal bipyramids, which is edge sharing with two of the tetrahedra.

#### Cd_4_SF_6_

Eight Cd(ii) atoms per unit cell adopt a distorted 8-fold coordination with Cl atoms. The S atom locates in monolayers in the *ab* plane, and the four Cd atoms that are adjacent to these layers are 7-coordinate with 3 neighbouring S and 4 neighbouring F neighbouring atoms. All of the polyhedra in the structure are edge sharing.

Having established promising compositions and their candidate structures, we next go on to perform quantitative analyses of the detailed electronic structure of these materials.

### II.VI. Optoelectronic properties

The most critical property for any light-harvesting material, whether for photovoltaic or solar fuel applications, is the bandgap (*E*_g_). Indeed, the screening procedure we have employed thus far relies on making initial estimates of *E*_g_ at an early stage, before considering structure or stability. The calculations required to accurately predict bandgaps are significantly more computationally demanding than those which can satisfactorily predict equilibrium geometry.

The first-principles values of *E*_g_ are presented in Table 2 alongside the bandgaps estimated using the SSE scale. Two of the compounds found by the screening procedure, Cd_5_S_4_Cl_2_ and Cd_4_SF_6_, have bandgaps in the visible range of 2.75 and 2.15 eV, respectively. Sn_5_S_4_Cl_2_ has a bandgap of 0.9 eV, which is better suited for solar cell or thermoelectric applications. This is encouraging, given the small set of compounds that have been brought through to this stage of the screening process and the qualitative nature of the SSE metric employed to screen the bandgaps.

**Table 2:** The parent-structure formulae from the ICSD compounds identified by analogy that led to the lowest energy structures after DFT relaxation are shown along with the energies above the convex hull (*E*analogyhull), and the corresponding energies predicted after global structure search (*E*globalhull). The estimated bandgaps from SSEs (*E*SSEg) used at the beginning of the workflow, bandgaps (*E*_g_), electron affinities (EA) and ionisation potentials (IP) calculated using a hybrid exchange-correlation functional at the end of the screening workflow, and effective masses for carrier electrons and holes from GGA calculations (
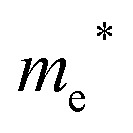
 and 
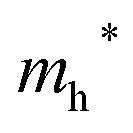
) are also displayed

Compound	Parent	*E* analogy hull (meV per atom)	*E* global hull (meV per atom)	*E* SSE g (eV)	*E*_g_ (eV)	EA (eV)	IP (eV)	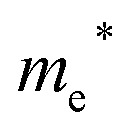	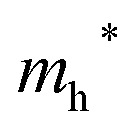
Sn_5_S_4_Cl_2_	Hg_5_ (O_2_Cl)_2_	96.5	61.8	2.0	0.91	3.30	4.21	0.50	0.40
Sn_4_SF_6_	Hg_4_OF_6_	51.8	46.7	2.0	3.36	2.45–2.94^*a*^	5.81–6.30^*a*^	0.86	2.01
Cd_5_S_4_Cl_2_	Hg_5_ (O_2_Cl)_2_	83.5	50.2	1.9	2.75	3.33	6.08	0.18	2.58
Cd_4_SF_6_	Cd_4_F_6_O	18.2	18.0	1.9	2.15	4.33	6.48	0.25	2.00

^*a*^When only polar surfaces could be found, a dipole correction term was added to the calculation of the surface dipole, which yields upper and lower bounds to the EA and IP values (see Computational methods section).

**Fig. 5:**
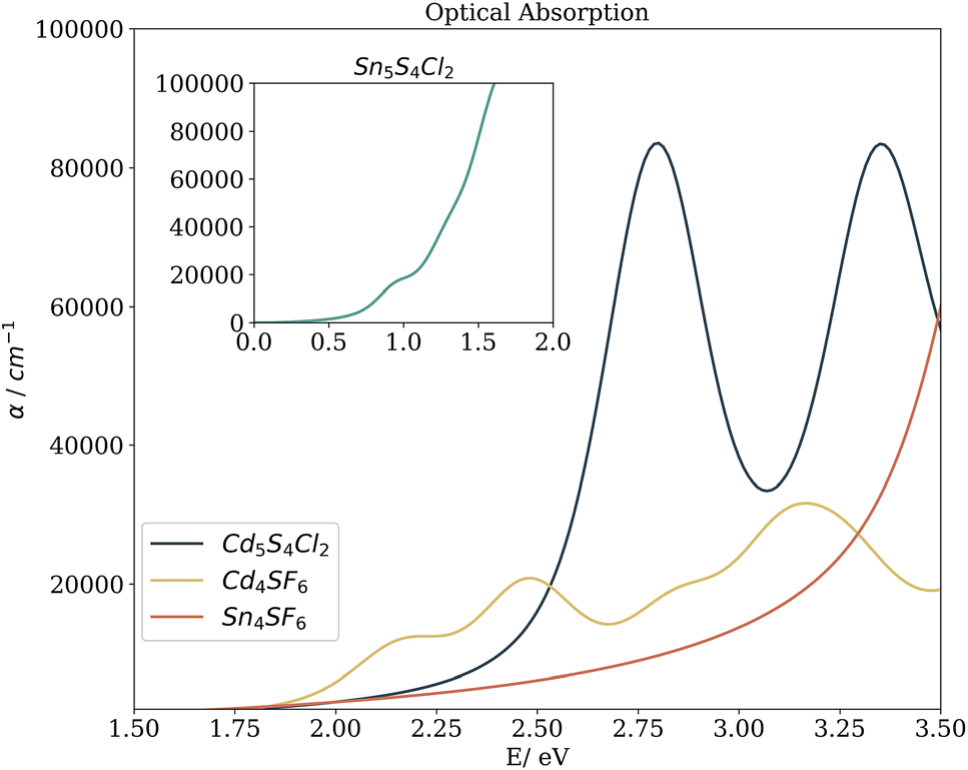
Simulated optical absorption spectra of the candidate materials from the complex dielectric function. Calculations were performed within DFT and the non-local HSE06 exchange-correlation functional, using the independent particle approximation (excluding excitonic and phonon-assisted transitions).

Beyond the bandgap, quantum-mechanical calculations can also provide access to optical absorption spectra *via* computation of the complex dielectric function. Fig. 5 shows the simulated spectra of the four compounds of interest. The Cd compounds display moderate absorption in the visible region, indicating their potential for use as solar fuel or photovoltaic materials. Of the two, Cd_4_SF_6_ absorbs photons with energy across more of the visible range but quite weakly, suggesting that thicker layers would be needed in a device. Meanwhile, Cd_5_S_4_Cl_2_ absorbs more strongly but at a higher energy, so would be suited to incorporation into a tandem solar cell.

**Fig. 6:**
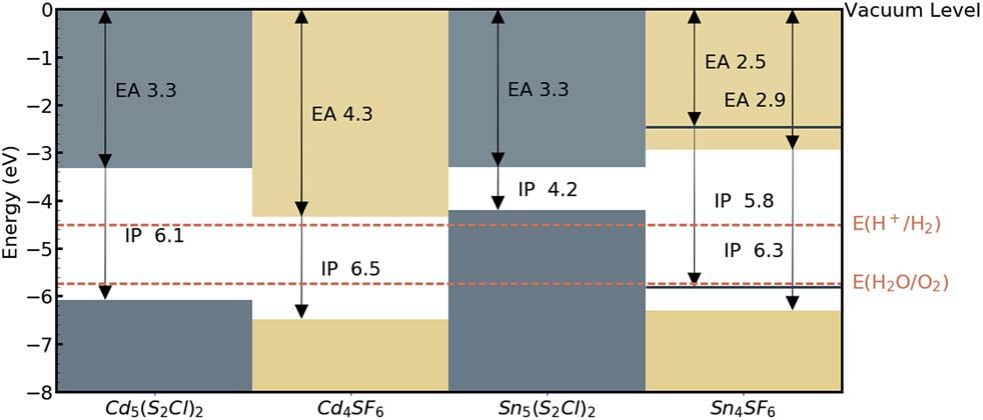
Electron affinities (EA) and ionisation potentials (IP) for the candidate materials, from DFT calculations of non-polar crystal terminations. The water redox potentials (dashed orange lines) are also shown. For Sn_4_SF_6_, a dipole correction was added resulting in lower and upper (blue solid lines) bounds for the IP and EA values.

The absolute band edge positions are also calculated using surface (non-polar slab) models of the four materials. The CBM position is the negative of the electron affinity (EA), and as indicated in Table 2, the EA values are all <4.5 eV. This indicates that as well as having promising bandgaps, the two Cd-based compounds have potential for use in photoelectrochemical water splitting applications, with VBM and CBM positions that bridge the water oxidation and reduction potentials, enabling the redox reaction. For Sn_4_SF_6_, no slab without an overall dipole could be found, so we instead report a likely range for the EA and IP values after applying a dipole correction in the slab calculation (see Computational methods section). This material also bridges these energies, but has too wide a band gap, while the other Sn-containing compound, Sn_5_S_4_Cl_2_, has an appropriate EA, but too small a bandgap, as has already been discussed. This is summarised in the energy band alignment diagram, Fig. 6.

**Fig. 7:**
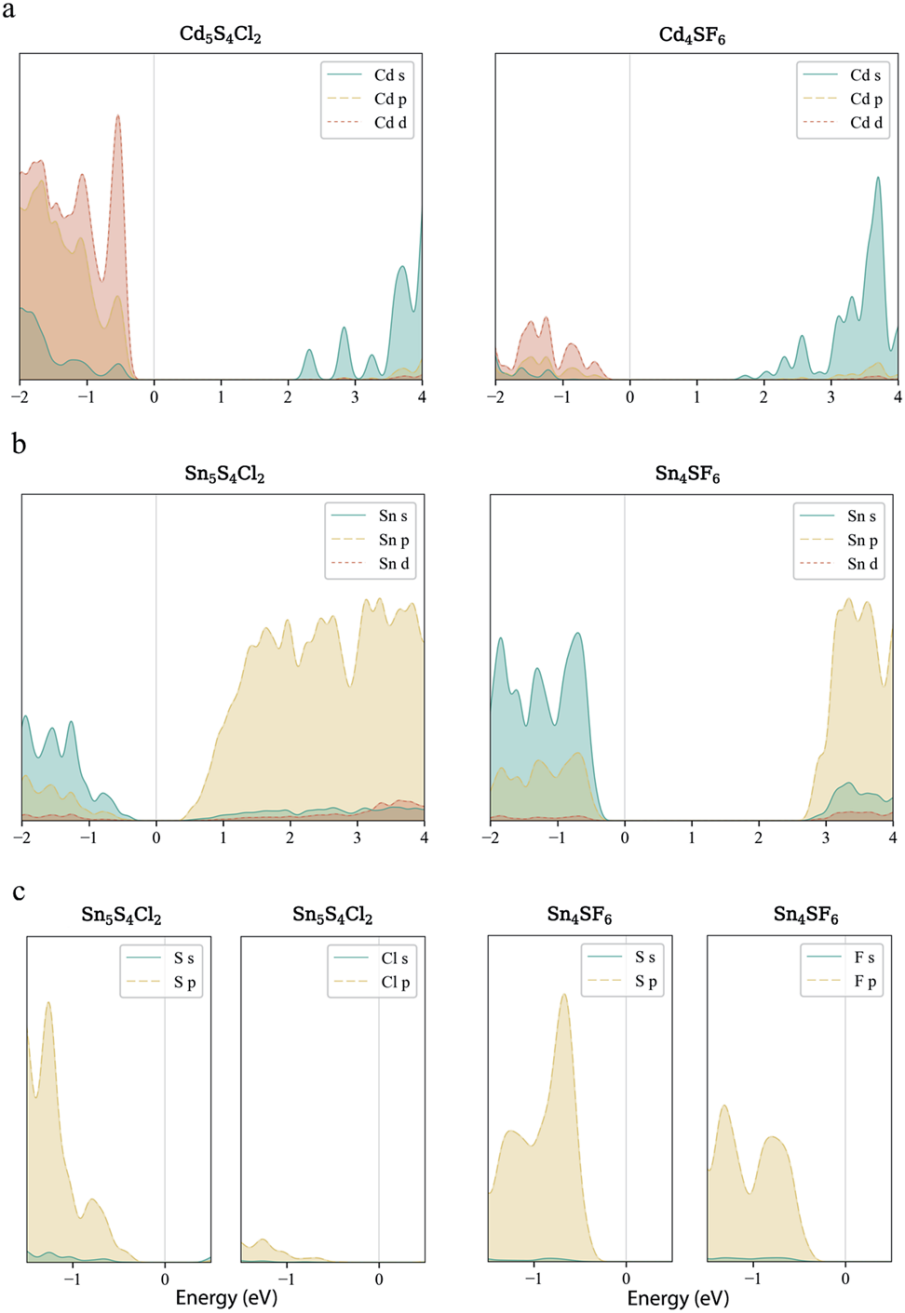
Orbital-projected local electronic density of states of Cd_5_S_4_Cl_2_, Cd_4_SF_6_, Sn_5_S_4_Cl_2_ and Sn_4_SF_6_. s- p- and d-orbital contributions from the metal species to the density of states near the band edges for the Cd-containing (a) and Sn-containing (b) compounds. The s- and p-orbital contributions from S and the halide species to the upper valence band for the Sn-containing compounds are also shown (c).

Finally, carrier effective mass (m*) is a quantity that can also provide preliminary insight into the performance of a semiconducting material, with smaller m* values being more desirable as this quantity is inversely proportional to conductivity. The two Cd-containing compounds have lower 
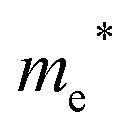
 values than the Sn-containing compounds (Table 2). This is a result of the metallic s-states forming the lower conduction band in the former case which give higher dispersion than the more directional metallic p-states in the latter (Fig. 7a and b). The 
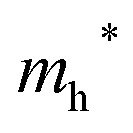
 values are in general much higher, with the sulphur and halide p-states dominating the upper valence band. One notable exception is Sn_5_S_4_Cl_2_ with a value of 0.40 *m*_e_. This is a result of strong hybridisation between the Sn s and S p orbitals which form a two-dimensional Sn–S network along which carriers can transport without encountering a Cl atom (Fig. 4). This is possible due to the Sn^2+^ oxidation state, which results in the Sn s orbitals remaining occupied. In the case of Sn_4_SF_6_, no such Sn–S network exists and S p states dominate the VBM, while F p states also contribute (Fig. 7c).

The calculated band structure of Sn_5_S_4_Cl_2_ reveals the presence of multiple band extrema (“multi-valley”), a sought-after feature in the design of thermoelectric materials.^48^ Furthermore, the effective number of extrema is increased by the presence of multiple bands within a few *k*_B_*T* in energy of each other at the *R*, *T*, *S* and *U* points in the Brillouin zone (see ESI Fig. S4†).

## III. Conclusion

We have introduced a hierarchical screening procedure and used it to search through a large space of over 161 000 compositions to identify promising candidate photoactive semiconductors. Using our approach, which relies on compositional descriptors and exploits existing data, first-principles calculations were carried out on a subset of compounds in order to establish thermodynamic stability, and global structure searching was employed for the most promising candidates. This procedure has enabled us to identify four new chalcohalide compounds, two of which, Cd_5_S_4_Cl_2_ and Cd_4_SF_6_, have bandgaps in the visible range and good absorption properties for solar fuel applications. Further detailed investigation into the electronic structure of these materials show that effective electron and hole conduction should be possible. The approach constitutes a computer-aided materials design procedure that employs existing knowledge in a targeted manner in order to traverse the vast chemical hyperspace.

## IV. Computational methods

### IV.I. Compositional screening

Construction of the search space and subsequent screening based on SSE and HHI_R_ is carried out with Python 3 on a desktop computer using the smact library, which is publicly available online at ; http://github.com/WMD-group/SMACT. First, the compositional search space of ternary chalcohalides is constructed using the smact package: the stoichiometry maximum is set to 8 and only those compositions which pass both the charge neutrality and electronegativity balance tests form part of the initial search space. Every possible combination of A_*x*_B_*y*_C_*z*_ is generated with B = [O, S, Se, Te] and C = [F, Cl, Br, I]. All known oxidation states of all elements in each combination are considered and charge neutrality is assessed by

1 *xq*_A_ + *yq*_B_ + *zq*_C_ = 0

where *q* is the formal charge associated with each species in the considered oxidation state. Combinations satisfy electronegativity balance when *χ*^cation^ < *χ*^anion^, where *χ* is the Pauling electronegativity^49^ of an element. This ensures the most electronegative elements carry the most negative charge. For full details of this method of search space construction, the reader is referred to ref. 30.

The SSE scale^34^ is used to limit the A cations to those with a SSE higher than the water reduction potential and the bandgap window was set to 1.5–2.5 eV. The SSE provides information on valence and conduction bands on the basis of the Frontier orbitals of the constituent ions. It reflects ionisation potential of an anion (filled electronic states) and electron affinity of a cation (empty electronic states). The energies of 40 elements were originally fitted from a test set of 69 closed-shell binary inorganic compounds, and now the SSE values for 94 elements are available.^50^ The bandgap (*E*_g_) can then be estimated from the tabulated SSE values as

2 *E*SSEg = SSE^cation^ – SSE^anion^

For multicomponent systems, the limiting SSE values are used.

### IV.II. Crystal structure prediction by analogy

We use the structure substitution algorithm developed by Hautier *et al.*,^40^ as implemented in the Pymatgen framework^41^ with a probability threshold of 0.001. For a given composition the procedure is carried out for each common oxidation state of the metal (*e.g.* for Sn_*x*_s_*y*_Cl_*z*_ both Sn(ii) and Sn(iv) must be considered).

### IV.III. Crystal structure prediction by global searching

Global crystal structure searches are carried out for each of the candidate compositions using the same stoichiometries as the lowest energy crystal structures from the prediction by analogy. This step is only carried out if a structure found by analogy falls within the defined “metastability window” of 100 meV per atom. Using the evolutionary structure prediction algorithm uspex,^45^,^46^ we perform global structure searches for the candidate compositions. No constraints are imposed on the shape or volume of the unit cell, but the search is restricted to one (11 atoms per cell) and two (22 atoms per cell) formula units for each of the four compositions. In the evolutionary optimisation procedure, the first generation contains 80 randomly generated structures, and the succeeding generations (each with 60 structures) are produced by random (20%), heredity (50%), permutation (10%), soft-mutation (10%), and lattice mutation (10%) operations as described elsewhere.^46^

### IV.IV. First-principles calculations

All first principles calculations are carried out using Kohn–Sham DFT with a projector-augmented plane wave basis^51^ as implemented in the Vienna *Ab initio* Simulation Package (vasp).^52^,^53^

#### Total energies

For calculating *E*_hull_ we use the PBEsol exchange-correlation functional.^43^ A Monkhorst–Pack *k*-point grid is generated for each calculation with *k*-point spacing of 0.242 Å^–1^. The kinetic-energy cutoff is set at 520 eV and the force on each atom converged to within 0.005 eV Å^–1^. The Materials Project API^54^ is used to retrieve DFT total energies of known phases for each chemical system. Phase diagrams are constructed to identify decomposition products and the total energies of these products recalculated in the same manner as described above.

#### Dynamical stabilities

Structures are further relaxed using a kinetic energy cutoff of 700 eV. The normal modes are calculated within the harmonic approximation, using the PHONOPY package^55^–^57^ to construct and evaluate the force constants. The finite displacement method (FDM) approach is used with a step size of 0.01 Å. Each of the unit cells contains *N* atoms (where *N* = 22 or 33) so has 6*N* (132 or 198) possible displacements. The number of unique displacements is reduced to between 11 and 44 depending on the crystal symmetry. For computational efficiency, phonons are considered at the *Γ* point only.

#### Optoelectronic properties

Semi-local exchange-correlation treatments such as the PBEsol functional provide an accurate description of crystal structures but tend to underestimate the electronic bandgaps of semiconductors. To overcome this issue, more accurate electronic structure calculations are performed using the hybrid non-local functional HSE06,^58^ which includes 25% screened Hartree–Fock exact exchange. *Γ*-centred homogeneous *k*-point meshes are used, the spacings of which are determined by the magnitude of the lattice vectors, as per Yu *et al.*^59^ and the kinetic energy cutoff is set at 520 eV. For optical absorption calculations, the dielectric tensor is calculated using the vasp code following the Kubo–Greenwood method. This is then used to calculate the absorption *via* the Kramers–Kronig relation.

Absolute electron energies (IP and EA values) are calculated by generating 2D slab models of low Miller index, non-polar surfaces of the crystal structures. Hybrid DFT (HSE06 functional) is used to calculate the surface dipole, *D*, which is the difference between the average electrostatic potential in the slab and that in the vacuum level. The VBM and CBM positions from the bulk calculations can then be used to calculated the true VBM and CBM positions. These are simply the differences between *D* and VBM_bulk_, and *D* and CBM_bulk_, respectively. Convergence with respect to slab thickness and vacuum distance was achieved within two repeat layers and 15 Å respectively, in all cases. When no non-polar surfaces could be found for a material, a dipole–dipole correction, as implemented in the vasp code, was added to the potential. This leads to an upper and lower limit of the potential in the vacuum level, and hence an upper and lower limit to *D*.

Carrier effective masses are calculated using band structures generated from hybrid DFT (HSE06 functional) calculations. The SeeKpath code^60^ is used to generate a suitable path through the Brillouin zone, which is sampled at a resolution of 0.01 Å^–1^ between each *k*-point. In order to calculate effective masses, a parabola is fit to all points from the minimum (maximum) of the CBM (VBM) to the points *k*_B_T higher (lower).

## V. Data access statement

The smact package can be accessed from ; https://github.com/WMD-group/SMACT. Screening results from these calculations may be reproduced using the Python code available on-line from ; https://github.com/WMD-group/SMACT/tree/master/examples. Optimised structures are available on-line from ; https://github.com/WMD-group/Crystal_structures/Chalcohalides. All other data may be obtained from the authors on request.

## Conflicts of interest

There are no conflicts to declare.

## Supplementary Material

Supplementary information
