# Supplementary material for: Computer-aided design of metal chalcohalide semiconductors: from chemical composition to crystal structure
Source: Chem Sci. 2017 Dec 4;9(4):1022–30. doi: 10.1039/c7sc03961a (PMC5883896; doi:10.1039/c7sc03961a)
Supplement: Supplementary file 1 [file SC-009-C7SC03961A-s001.pdf]

Electronic Supplementary Material (ESI) for Chemical Science.
This journal is © The Royal Society of Chemistry 2017

Design of Photoactive Metal Chalcohalide Semiconductors: From Composition to Structure with Data-mining and Global Optimisation

Daniel W. Davies,[1] Keith T. Butler†,[1] Jonathan M. Skelton,[1] Congwei Xie,[2] Artem R. Oganov,[3,4,5] and Aron Walsh[6,7]

[1] *Centre for Sustainable Chemical Technologies and Department of Chemistry, University of Bath, Claverton Down, Bath BA2 7AY, UK*

[2] *Science and Technology on Thermostructural Composite Materials Laboratory, International Center for Materials Discovery, School of Materials Science and Engineering, Northwestern Polytechnical University, Xian, Shaanxi 710072, Peoples Republic of China*

[3] *International Center for Materials Discovery, School of Materials Science and Engineering, Northwestern Polytechnical University, Xian, Shaanxi 710072, Peoples Republic of China*

[4] *Department of Geosciences, Center for Materials by Design, and Institute for Advanced Computational Science, State University of New York, Stony Brook, New York 11794-2100, USA*

[5] *Moscow Institute of Physics and Technology, Dolgoprudny, Moscow Region 141700, Russia*

[6] *Global E^3 Institute and Department of Materials Science and Engineering, Yonsei University, Seoul 120-749, Korea*

[7] *Department of Materials, Imperial College London, Exhibition Road, London SW7 2AZ, UK*

(Dated: 15 November 2017)

I. SUPPLEMENTAL DATA ITEMS

A. Dynamic Stability

No negative frequency phonon modes were found at Γ for the structures predicted by global optimisation. The phonon densities of states are shown in Figure S1. The negative frequency phonon modes in the $Sn_5S_4Cl_2$ structure are at the Y, T and Z high symmetry points in the Brillouin zone. These imaginary modes would not be present if the structure was doubled along X (and Y as these are equivalent) and T were considered. Due to the practical limits of the size of the unit cell that can be considered for a global search, such a structure was not identified.

For the compounds predicted by analogy, the $Cd_5S_4Cl_2$ and Cd_4SF_6 structures both had modes with negative frequencies (imaginary modes) which indicate a lack of a restoring force when a ions are displaced along the collective mode coordinate. Although this can often indicate dynamical instability, mapping out of the modes in question can in each case provide a satisfactory explanation for their presence.

In the case of the $Cd_5S_4Cl_2$ structure, three imaginary modes were found. Mapping of the first reveals a double well potential energy surface (Figure S3a). The second and third reveal two extremely shallow degenerate double wells (Figure S3b). The structure did not relax into one of these wells during the DFT relaxation step due to limitations inherent to the numerical optimisers used for structure relaxation; if the structure is at a saddle point with some symmetry on the potential-energy surface, it is unlikely that the optimiser will break the symmetry to find a minimum, as the forces on the structure are balanced. Nudging the structure into the larger of the two wells in Figure S3a results in a slight reduction of total energy and elimination of all three imaginary phonon modes. Mapping of the one imaginary mode present for the Cd_4SF_6 structure reveals a wide, flat-bottomed potential (Figure S3c), which suggests the imaginary mode is due to anharmonicity rather than the system being a saddle point on the energy surface.

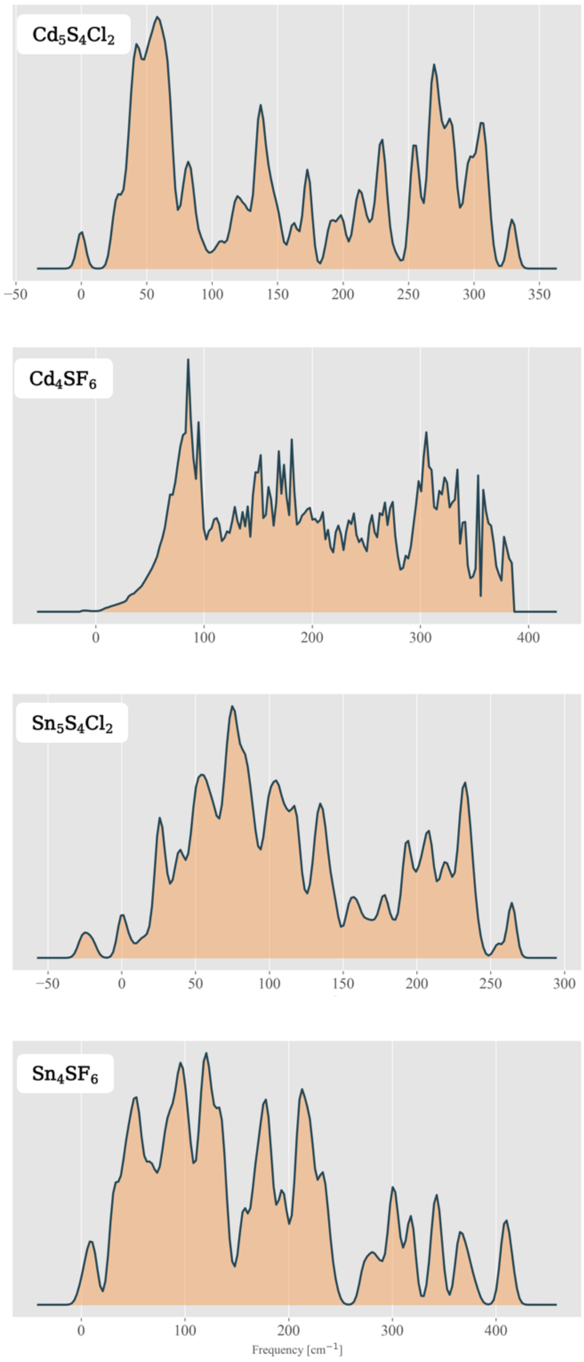

FIG. S1: Phonon densities of states for each of the structures found by global optimisation.

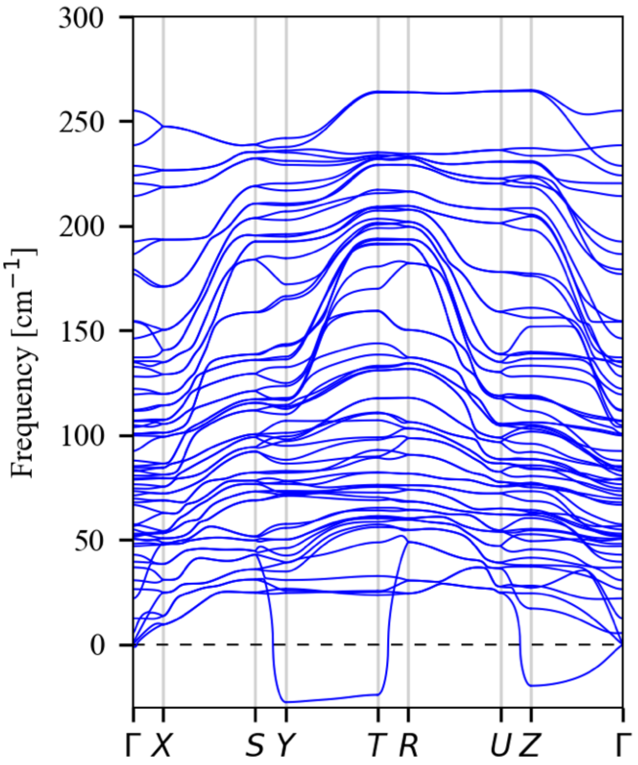

FIG. S2: Phonon band structure from a $1 \times 2 \times 2$ supercell of the $Sn_5S_4Cl_2$ crystal structure found by global searching.

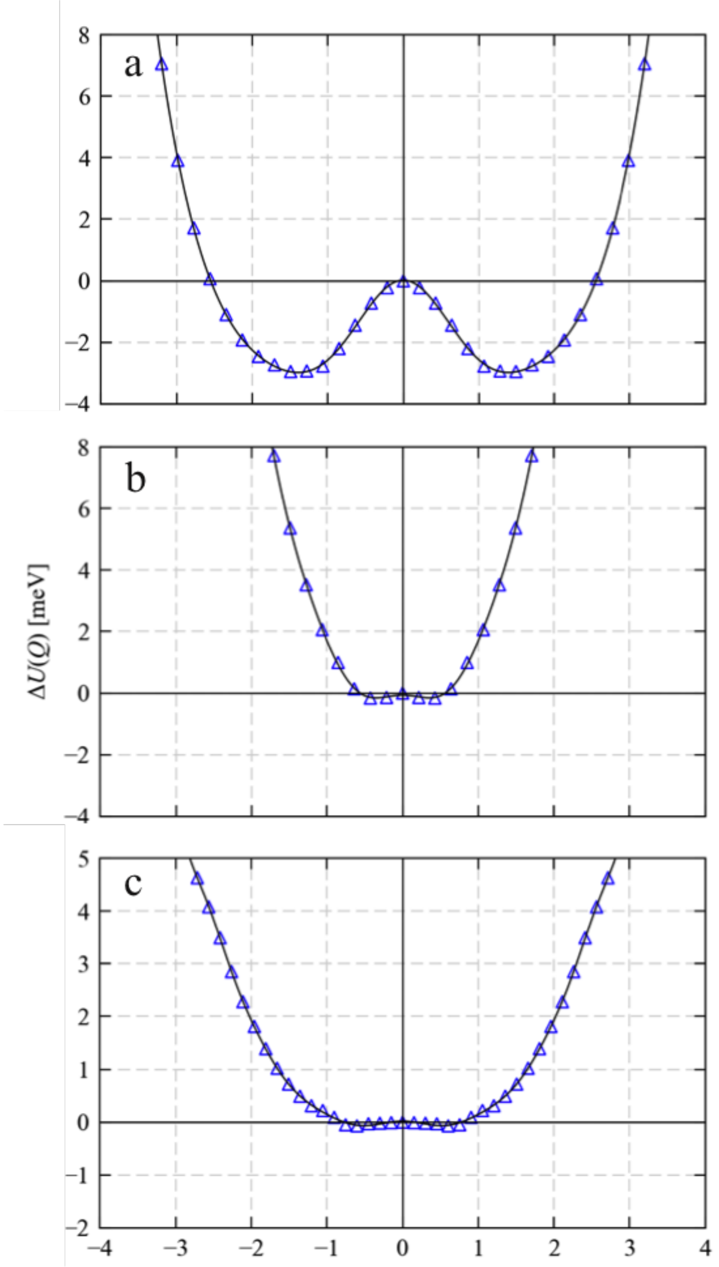

FIG. S3: Potential energy mapping of the imaginary phonon modes in the $Cd_5S_4Cl_2$ (a and b) and Cd_4SF_6 structures (c) found by analogy with known structure types.

B. Electronic Band Structures

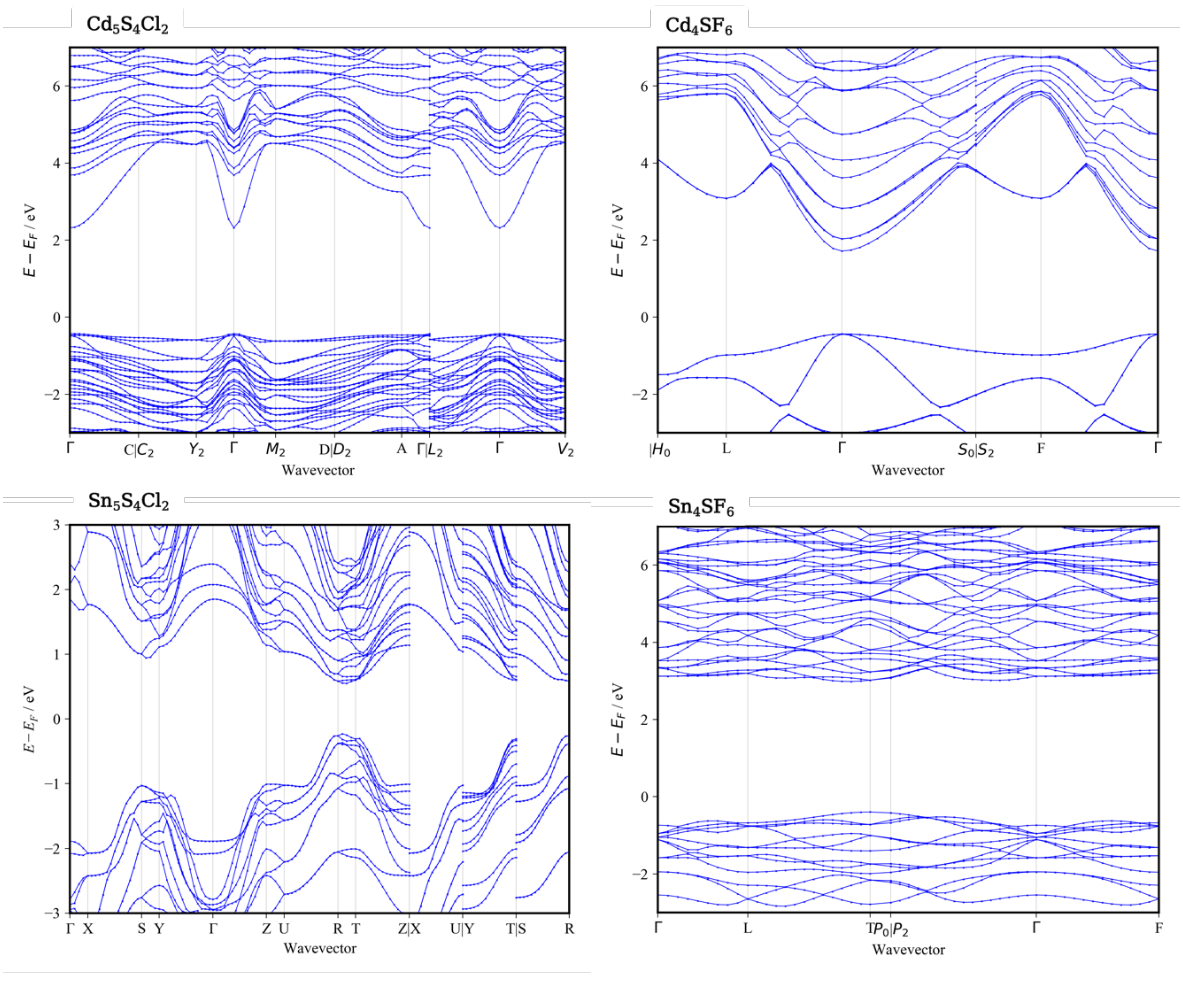

FIG. S4: Electronic band structures of the proposed chalcohalide compounds calculated using DFT and the HSE06 hybrid functional.
